# Genetically predicted higher educational attainment decreases the risk of stroke: a multivariable Mendelian randomization study

**DOI:** 10.1186/s12872-022-02713-7

**Published:** 2022-06-16

**Authors:** Weihao Zhang, Yuanjin Li, Yuming Li, Kai Zheng, Shenghui Zou, Xing Jia, Hua Yang

**Affiliations:** 1 Department of Neurosurgery, The Affiliated Hospital of Guizhou Medical University, Guizhou Medical University, Guizhou, 610041 China; 2 Department of Plastic Surgery, The Third People’s Hospital of Guizhou Province, Guizhou, 610041 China; 3 Department of Neurosurgery, People’s Hospital of Jiajiang County, Leshan, 614000 Sichuan China

**Keywords:** Causal association, Educational attainment, Mendelian randomization, Stroke, Single nucleotide polymorphisms

## Abstract

**Background:**

The causal association between educational attainment (EA) and stroke remains unclear. Hence, a novel multivariable Mendelian randomization (MVMR) approach was applied to solve this issue.

**Methods:**

The single nucleotide polymorphisms (SNPs) from a recent genome-wide association study (GWAS) on years of schooling served as instruments. Univariable mendelian randomization (MR) and MVMR analyses were performed to detect the relationship between genetically predicted EA and the stroke risk. In the MVMR, cigarette consumption, alcohol consumption, body mass index (BMI), intelligence, and hypertension were adjusted. The summary statistics for stroke from the MEGASTROKE consortium included 446,696 participants (40,585 cases of stroke and 34,217 cases of ischemic stroke), most of whom were of European descent.

**Results:**

In the univariable MR, genetically predicated EA could decrease the risks of total stroke (OR = 0.66, 95% CI 0.61–0.72, *P* = 2.70 × 10^–23^), ischemic stroke (OR = 0.67, 95% CI 0.61–0.73, *P* = 2.58 × 10^–18^), large artery atherosclerosis (OR = 0.51, 95% CI 0.40–0.64, *P* = 1.80 × 10^–8^), small vessel stroke (OR = 0.60, 95% CI 0.49–0.73, *P* = 5.59 × 10^–7^), and cardioembolic stroke (OR = 0.81, 95% CI 0.68–0.96, *P* = 1.46 × 10^–2^) using the inverse-variance weighted (IVW) estimator. Higher EA might be negatively correlated with the odds of total stroke (OR = 0.62, 95% CI 0.50–0.77, *P* = 1.44 × 10^–5^), ischemic stroke (OR = 0.63, 95% CI 0.50–0.80, *P* = 1.41 × 10^–4^), and cardioembolic stroke (OR = 0.59, 95% CI 0.39–0.90, *P* = 0.01), but was not significant in large artery atherosclerosis (OR = 0.65, 95% CI 0.37–1.15, *P* = 0.14) and small vessel stroke (OR = 0.68, 95% CI 0.41–1.13, *P* = 0.14) after controlling other exposures.

**Conclusions:**

We found that genetically predicated higher EA decreased the risks of total stroke, ischemic stroke, and cardioembolic stroke, independent of smoking, alcohol consumption, BMI, intelligence, and hypertension.

## Background

The definition of stroke has been updated as an acute episode of focal dysfunction of the brain, retina, or spinal cord, persisting ≥ 24 h, according to the American Stroke Association [1]. If imaging examination or autopsy shows related focal infarction or hemorrhage, stroke duration exceeding 24 h is not a requisite condition [1]. Currently, stroke is prevalent globally and heavily burdens society. As summarized by the Global Burden of Disease (GBD) Stroke Experts Group, the absolute numbers of stroke patients, stroke survivors, related deaths, and disability-adjusted life-years (DALYs) are excessive and still increasing [2]. It is noteworthy that stroke has been the second leading cause of death [3]. Interventions before the onset of stroke seem requisite and may reduce its growing incidence and achieve a better prognosis.

Heterogeneous risk factors for stroke have been identified in previous publications. Cardiovascular factors (including hypertension, carotid stenosis, and atrial fibrillation), metabolic factors (comprising dyslipidemia, insulin resistance, and diabetes) and modifiable lifestyles (such as cigarette and alcohol consumption) have been found to increase stroke risks [3]. In addition, education is reported to be negatively associated with some stroke subtypes, which may protect the general population from a perspective of epidemiology. Wen et al. reported that participants with higher educational attainment (EA) had lower risks of incident total stroke and ischemic stroke (IS) in a prospective cohort enrolling 11,509 participants. Nevertheless, the same findings were not found in hemorrhagic stroke [4]. For IS, the risk of recurrent stroke increased 2.82-fold for illiterate in a two-year follow-up duration [5]. However, in a follow-up study with 253,657 participants conducted by Jackson CA et al., EA was not associated with the increased risks of stroke in a fully adjusted model [6]. These inconsistent findings may be attributed to the observational design, which cannot overcome the endogeneity and the biases from confounding factors. A clear, unbiased estimate between EA and stroke using multivariable Mendelian randomization (MVMR) is needed.

Mendelian randomization (MR) is an epidemiological method that studies the causal association between and exposure (i.e., educational levels) with an outcome (i.e., stroke), using genetic variants as instruments to infer levels of the exposure. [7]. The genetic variants, closely related to the exposures and outcomes, are identified using the genome-wide association study (GWAS) and randomly assorted at conception, leading to a subsequent random distribution [8]. These randomly assorted variants avoid the reversed causation and confounding factors (i.e., smoking, diabetes, and alcohol), allowing for causal inference [9]. Therefore, a natural randomized control trial is simulated using the MR method. Moreover, MVMR is an extension that can produce the causal estimates of several exposures to one outcome, which is advantageous in the presence of several correlated risk factors, accounting for the measured pleiotropy [10].

In this study, MVMR was adopted to overcome the endogeneity and yield causal estimates between EA and stroke after controlling for smoking, alcohol consumption, BMI, intelligence, and hypertension. Although similar MR exploring the causal effects of EA on stroke was done by Yuan et al. in the past [11], the present study relies on the use of the MVMR design to test the effect of confounders in the association between EA and stroke. This study can help clarify the current inconsistent findings between EA and stroke.

## Methods

### Genetic instrument selection for EA

The GWAS summary dataset for EA was extracted from Social Science Genetic Association Consortium (SSAGC) Data Portal (http://thessgac.com). Using meta-analysis, GWAS combined 71 cohort studies, including 1,131,881 samples of European ancestry. The survey collected the years of schooling of participants. Detailed information regarding phenotype and the process of quality control in SSAGC was reported in a previous paper [12]. The summary statistics without 23andMe were obtained from the SSAGC consortium.

We included autosomal biallelic SNPs with a *P*-value < 5 × 10^–8^ and conducted further quality control based on a minor frequency > 1%, leaving 30,389 unique SNPs. In addition, using the 1000G reference panel, linkage disequilibrium (LD) clumping was performed. A total of 30,389 SNPs were clumped with LD *r*^2^ < 0.01 at a 10,000 kb window to guarantee the independence of the selected genetic variants.

Finally, 481 independent SNPs were associated with EA. The proportion of variance explained (PVE) by each SNP was estimated using the *R*^2^ value. The instrumental strength of each SNP was assessed using *F*-statistics through the formula: *F*-statistics = (Beta/Se)^2^. We are reporting the mean *F-statistic* of the SNPs used as instruments, while an *F-statistic* > 10 indicated a strong instrument [13].

### Genetic instrument selection for stroke

The summary statistics for stroke, IS and IS subtypes (e.g., large artery stroke, small vessel stroke, and cardioembolic stroke) were obtained from the MEGASTROKE consortium [14]. The IS subtypes were 4,373 cases of large artery atherosclerosis, 5,386 cases of small vessel stroke, and 7,193 cases of cardioembolic stroke. In the study, 40,585 stroke patients, 34,217 IS patients, and 406,111 controls were selected all of European population.

### Genetic instrument selection for other exposures

Other sources of GWAS are available from the IEU Open GWAS Project (http://gwas.mrcieu.ac.uk). Several exposures associated with EA and stroke are included in the MR analysis, such as smoking, alcohol consumption, intelligence, body mass index (BMI), and hypertension. According to Davies G et al., intelligence is closely related to education [15]. In addition, hypertension, obesity, smoking, and alcohol consumption are widely accepted as modifiable risk factors for stroke [16]. We tested if exposure to these five risk factors affect the association between EA and stroke using MVMR. The genetic data of intelligence, released by the Complex Trait Genetics (CTGlab), included 269,867 Europeans [17]. The genetic data on smoking and alcohol consumption were downloaded from the GWAS and Sequencing Consortium of Alcohol and Nicotine use (GSCAN), covering 335,394 and 337,334 sample sizes for tobacco use and alcohol intake, respectively [18]. Summary-level GWAS data on BMI were published in the Genetic Investigation of Anthropometric Traits (GIANT), and data on hypertension were summarized from the FinnGen biobank analysis round 5 (https://www.finngen.fi/fi) (Code: finn-b-I9_HYPTENSESS) [19, 20].

### Statistical analysis

In this study, the inverse-variance weighted (IVW) method was used as the main method to estimate the causal association of EA on stroke, IS, and IS types. The MR-Egger, weighted median, simple mode, and weighted mode were supplementary methods. The MR Pleiotropy Residual Sum and Outlier (MR-PRESSO) and MR-Egger intercept approaches were adopted to identify horizontal pleiotropy [21, 22]. The radial MR method was adopted to remove the potential outliers [23]. The Steiger-MR was used to examine SNPs that explained significantly more variance in exposure than the outcome and exclude those SNPs from the MR analyses to eliminate bias from reverse causation [24]. In this study, no inverse directionality was detected, and consequently, no SNPs were excluded in this step. The Bonferroni correction (*P* = 0.05/5 outcomes) was applied to adjust multiple testing (*P* = 0.01) in univariable MR. For the MVMR model, results of the IVW estimation were shown.

Univariable MR and MVMR methods were adopted using the R package "TwoSampleMR", and the results were visualized by the R package "forestplot". The data cleaning, statistical analyses, and visualization were run in R software 4.1.2 (https://www.r-project.org/).

All methods were performed following the strengthen the reporting of observational studies in the epidemiology using Mendelian Randomization (STROBE-MR) statement. All the summary-level GWAS data used in MR analyses are publicly accessible from the IEU Open GWAS Project database (http://gwas.mrcieu.au.uk). Informed consent was obtained from all subjects in the original genome-wide association studies. All methods were performed following the relevant local guidelines and regulations.

## Results

### Results of univariable MR

**Fig. 1:**
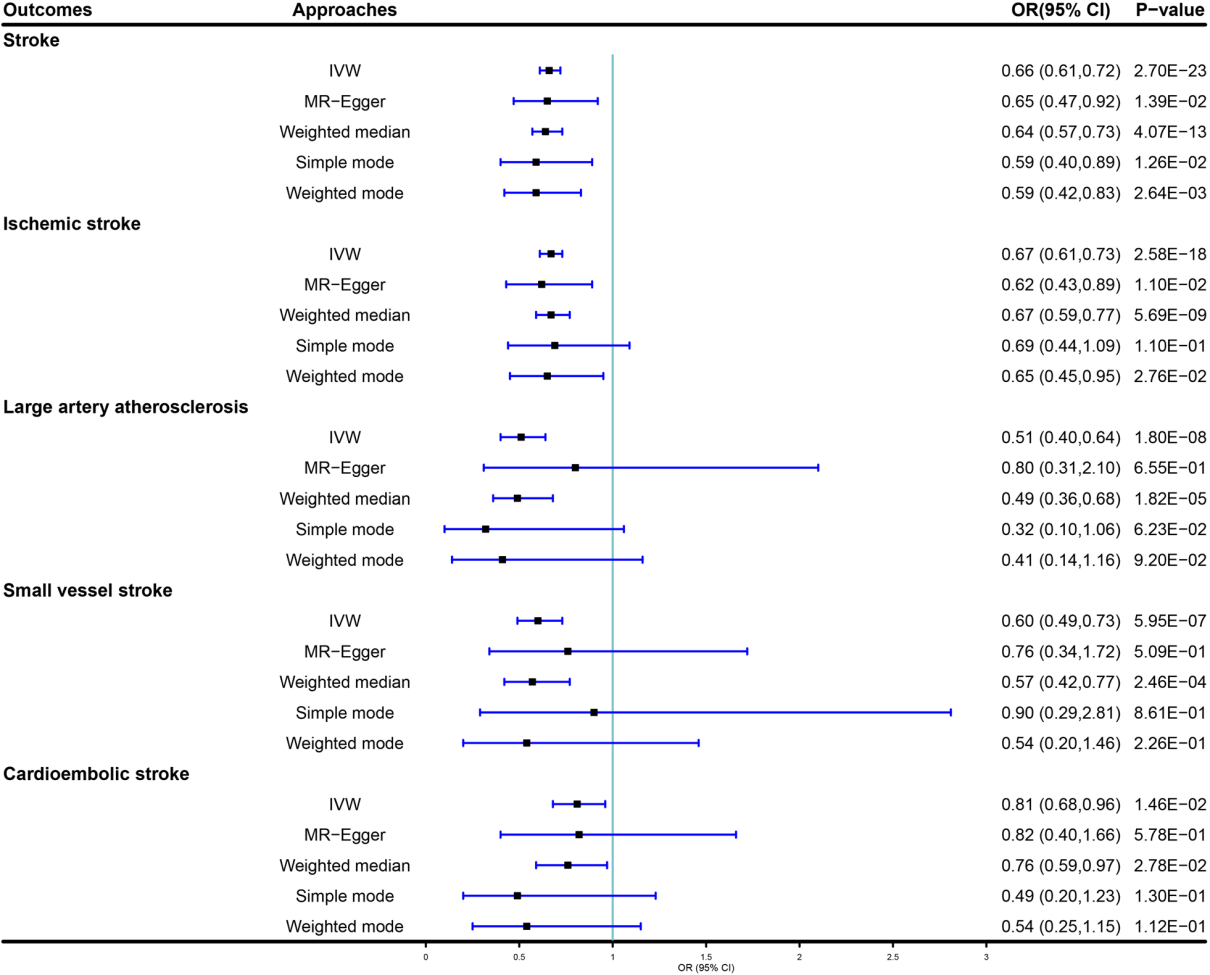
Causal estimates of EA on stroke in univariate MR. IVW: inverse variance weighted method; OR: odds ratios; EA: educational attainment; MR: Mendelian randomization

**Fig. 2:**
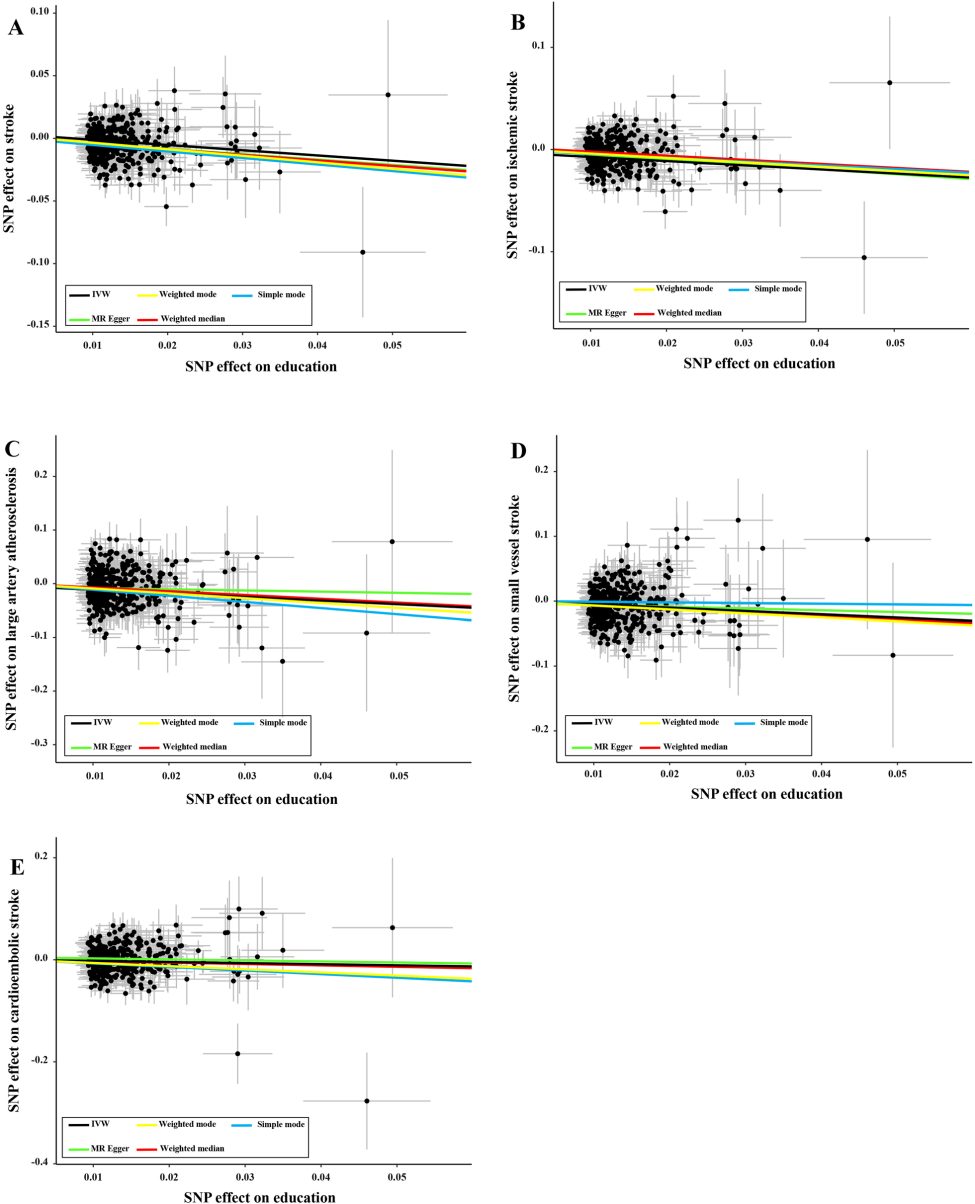
Scatter plots of EA on stroke. **A** Scatter plot of the effect size of each SNP on total stroke. **B** Scatter plot of the effect size of each SNP on ischemic stroke. **C** Scatter plot of the effect size of each SNP on large artery atherosclerosis. **D** Scatter plot of the effect size of each SNP on small vessel stroke. **E** Scatter plot of the effect size of each SNP on cardioembolic stroke. EA: educational attainment; IVW: Inverse variance weighted method; SNP: single nucleotide polymorphism

As shown in Fig. 1, in the univariable MR stage, each additional genetically predicted year of schooling can decrease the risk of stroke using the IVW estimator (OR = 0.66, 95% CI 0.61–0.72, *P* = 2.70 × 10^–23^). Similarly, MR-Egger and Weighted median yielded consistent direction of estimation, and they might have better statistical power than the IVW estimator (MR-Egger: OR = 0.65, 95% CI 0.47–0.92, *P* = 1.39 × 10^–2^; weighted median: OR = 0.64, 95% CI 0.57–0.73, *P* = 4.07 × 10^–13^). Additionally, each additional genetically predicted year of schooling could reduce the odds of IS using the IVW estimator (OR = 0.67, 95% CI 0.61–0.73, *P* = 2.58 × 10^–18^). Likewise, MR-Egger and Weighted median methods also reported similar results (MR-Egger: OR = 0.62, 95% CI 0.43–0.89, *P* = 1.10 × 10^–2^; weighted median: OR = 0.67, 95% CI 0.59–0.77, *P* = 5.69 × 10^–9^). For different IS types, each additional genetically predicted year of schooling was negatively associated with large artery atherosclerosis, small vessel stroke, and cardioembolic stroke (IVW: OR = 0.51, 95% CI 0.40–0.64, *P* = 1.80 × 10^–8^ for large artery atherosclerosis; OR = 0.60, 95% CI 0.49–0.73, *P* = 5.59 × 10^–7^ for small vessel stroke; OR = 0.81, 95% CI 0.68–0.96, *P* = 1.46 × 10^–2^ for cardioembolic stroke). The associations remain consistent in MR-Egger and weighted median. Figure 2 shows the scatter plots of univariable MR**.**

There were no evidence of heterogeneity and pleiotropy for EA with stroke, IS, large artery stroke, small vessel stroke, and cardioembolic stroke (MR-PRESSO global test and MR-Egger intercept: *P* > 0.05) (Table 1).

**Table 1:** Results of heterogeneity and pleiotropy tests

Outcomes	*P* values of MR-PRESSO	MR-egger intercept	Q-df value by IVW	Q-df value by MR-egger
Stroke	0.054^#^	8.20E−05^†^	411^†^	410^†^
Ischemic stroke	0.892^#^	1.05E−03^†^	409^†^	408^†^
Large artery atherosclerosis	0.001^#^	− 6.11E−03^†^	410^†^	409^†^
Small vessel stroke	0.712^#^	− 3.12E−03^†^	407^†^	406^†^
Cardioembolic stroke	0.053^#^	− 1.79E−04^†^	411^†^	410^†^

^#^Means no outliers were detected by the MR-PRESSO and radial MR methods; ^†^ means *P* > 0.05

### Results of multivariable MR

**Fig. 3:**
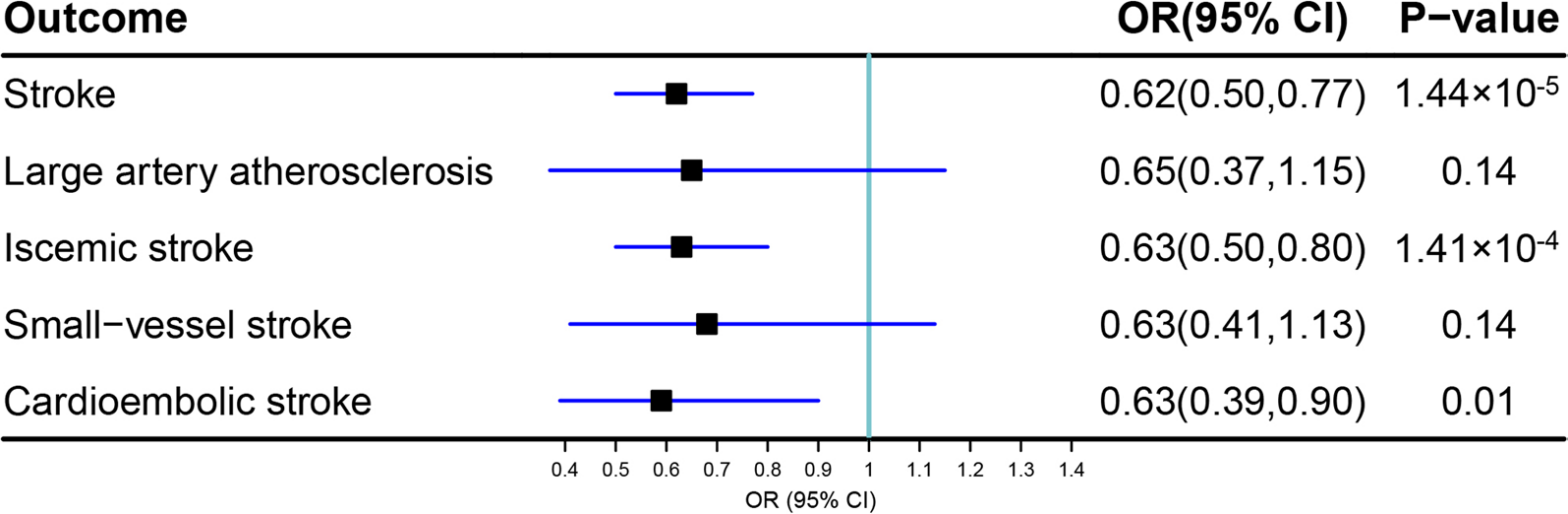
Causal estimates of EA on stroke in multivariate MR. Cigarette consumption, alcohol consumption, BMI, intelligence, and Hypertension were adjusted in the multivariate MR analyses. EA: educational attainment; MR: Mendelian randomization

As shown in Fig. 3, after controlling for smoking, alcohol consumption, intelligence, BMI and hypertension, each additional genetically predicted year of schooling was significantly associated with stroke, IS, and cardioembolic stroke using the IVW estimator (OR = 0.62, 95% CI 0.50–0.77, *P* = 1.44 × 10^–5^ for stroke; OR = 0.63, 95% CI 0.50–0.80, *P* = 1.41 × 10^–4^ for IS; OR = 0.59, 95% CI 0.39–0.90, *P* = 0.01 for cardioembolic stroke). The effects were not significant for large artery atherosclerosis (OR = 0.65, 95% CI 0.37–1.15, *P* = 0.14) after multiple testing correction. Instead, each additional genetically predicted year of schooling was not casually correlated with small vessel stroke (OR = 0.68, 95% CI 0.41–1.13, *P* = 0.14).

## Discussion

This MVMR study shows that higher EA causally decreases the risks of total stroke, IS, large artery atherosclerosis, and cardioembolic stroke except for small vessel stroke, independent of smoking, alcohol consumption, BMI, intelligence, and hypertension. These findings provide novel unbiased causal evidence supporting the protective role of education in stroke, which may help suppress the high stroke prevalence.

Many observational reports have mentioned the protective role of education in stroke [25, 26]. In a meta-analysis enrolling 79 studies, McHutchison CA et al. reported a 1.35-fold stroke risk in lower-education participants [27]. As revealed by Gillum RF et al. [28], compared with the participants with education years < 8, those with education years 8–11, 12, and > 12 had relative risks of 0.81, 0.57, and 0.60, respectively. Identical findings were also found in a study by Wen et al. [4], where participants with higher EA had lower risks of total stroke and IS during a median follow-up of 25.3 years [4]. However, a protective effect was not detected in males in their subgroup analysis. The discrepancy between males and females may be partly explained by gender features [29]. Despite the slight difference, these studies support the conclusion that higher EA is negatively associated with the incidence of stroke.

However, a negative association between education levels and stroke was not observed in other studies. In a large prospective study in Australia with a mean follow-up of 4.7 years, the fully adjusted hazard ratios of the lowest to highest education level in men and women were 1.10 (95% CI 0.94—1.30) and 1.21 (95% CI 0.97—1.51), respectively, which did not support the increased stroke risks for the lower EA [6]. These inconsistent findings may be attributed to the residual confounding factors, which can be limited by MR, such as alcohol and cigarette consumption. Our MVMR analysis can help clarify the unclear associations between education and stroke. Other MR studies also explored the causal association between EA and stroke. Yuan S et al. reported that EA decreased the risk of stroke, independent of intelligence, BMI, and smoking [11]. Their main findings were consistent with ours. However, in their study, two pivotal factors, alcohol consumption and hypertension, were not adjusted in the MVMR analysis, which might be the reason why the causal association of EA between large artery atherosclerosis and small vessel stroke was significant in Yuan S's study but not in our study.

Additionally, EA should be considered an intervention for the general population and especially stroke patients. A clear causal estimate between education and stroke helps ameliorate the current high stroke prevalence and poor prognosis after stroke. According to a follow-up study enrolling 3,861 Chinese by Che et al. [5], after developing IS, the hazard ratios of the illiterate to college education were 2.79 for all-cause mortality, 3.68 for stroke-specific mortality, 2.82 for recurrent stroke, and 3.46 for cardiovascular events.

The mechanisms linking EA to stroke remain unclear. And two possible ways may be involved in their causal association. First, the occurrence of stroke is not directly regulated by EA-associated genes, but was largely mediated by modifiable risk factors like blood pressure, BMI, and cigarette consumption [30]. Higher EA is generally associated with a healthier lifestyle, subsequently leading to the decreased risk of stroke. But on the other hand, Carter A R et al. also reports that more than half of the protective effect of higher EA are not attributed to the modifiable risk factors and remains unexplained [31]. Therefore, it is postulated that higher EA may suppress the occurrence of stroke directly, rather than through modifiable risk factors. As indicated by previous studies, the EA-associated molecular alteration in pathways involving inflammatory cytokines may be mediated by gene methylation, gene silencing etc. [32, 33]. Hence, the two ways may jointly link EA to stroke, but still need further confirmation in future studies.

There are some strengths and limitations in this study. The major merit lies in the MVMR design, which overcomes the endogeneity in observational studies and possible correlated risk factors in the univariable MR. However, genetic variants are identified in GWAS on participants of European descent, in order to avoid bias linked to ancestry, which limits the generalizability of our results. Further confirmation of other ancestries, such as Asian, should be performed. Additionally, pleiotropy and heterogeneity are two main concerns in MR analysis. In the two-sample MR analysis regarding large artery atherosclerosis and EA, MR-PRESSO reported a significant result of pleiotropy, indicating that the genetic variants used in this study may be associated with other confounding factors and violate the basic assumptions of MR. However, the weighted median method and multivariable MR results remain consistent with those of the IVW approach. The weighted median approach can yield consistent estimates even if up to 50% of the genetic instruments are invalid [34]. Considering the non-significant results of the MR-Egger regression, the bias may be minimal.

To conclude, this MVMR study provides new evidence supporting the protective role of higher EA in stroke. Intervention strategies to improve EA may have beneficial effects in individuals at high risk for stroke..

## Conclusions

Using MR, this study provides evidence of a causal association that higher EA decreases the risks of total stroke and IS subtypes except for small vessel stroke, independent of smoking, alcohol consumption, BMI, intelligence, and hypertension.

## Data Availability

The datasets generated and/or analyzed during the current study are publicly available in the IEU Open GWAS Project repository (http://gwas.mrcieu.au.uk).

## Abbreviations

BMI: Body mass index
CI: Confidence interval
CTGlab: Complex trait genetics
DALYs: Disability-adjusted life-years
EA: Educational attainment
GBD: Global burden of diseases
GIANT: Genetic investigation of anthropometric traits
GSCAN: GWAS & sequencing consortium of alcohol and nicotine use
GWAS: Genome-wide association studies
IEU: Integrative epidemiology unit
IS: Ischemic stroke
IVW: Inverse-variance-weighting
LD: Linkage disequilibrium
MVMR: Multivariable Mendelian randomization
OR: Odds ratios
PAGE: Population architecture using genomics and epidemiology
PVE: Proportion of variance explained
SNPs: Single nucleotide polymorphisms
SSAGC: Social science genetic association consortium
